# Extracellular Vesicles Produced by the Probiotic *Propionibacterium freudenreichii* CIRM-BIA 129 Mitigate Inflammation by Modulating the NF-κB Pathway

**DOI:** 10.3389/fmicb.2020.01544

**Published:** 2020-07-07

**Authors:** Vinícius de Rezende Rodovalho, Brenda Silva Rosa da Luz, Houem Rabah, Fillipe Luiz Rosa do Carmo, Edson Luiz Folador, Aurélie Nicolas, Julien Jardin, Valérie Briard-Bion, Hervé Blottière, Nicolas Lapaque, Gwenaël Jan, Yves Le Loir, Vasco Ariston de Carvalho Azevedo, Eric Guédon

**Affiliations:** ^1^INRAE, Institut Agro, STLO, Rennes, France; ^2^Laboratory of Cellular and Molecular Genetics, Institute of Biological Sciences, Federal University of Minas Gerais, Belo Horizonte, Brazil; ^3^Biotechnology Center, Federal University of Paraíba, João Pessoa, Brazil; ^4^INRAE, AgroParisTech, Paris-Saclay University, Micalis Institute, Jouy-en-Josas, France

**Keywords:** extracellular vesicles, membrane vesicles, probiotic, propionibacteria, immunomodulation, anti-inflammatory, IL-8, NF-κB

## Abstract

Extracellular vesicles (EVs) are nanometric spherical structures involved in intercellular communication, whose production is considered to be a widespread phenomenon in living organisms. Bacterial EVs are associated with several processes that include survival, competition, pathogenesis, and immunomodulation. Among probiotic Gram-positive bacteria, some *Propionibacterium freudenreichii* strains exhibit anti-inflammatory activity, notably via surface proteins such as the surface-layer protein B (SlpB). We have hypothesized that, in addition to surface exposure and secretion of proteins, *P. freudenreichii* may produce EVs and thus export immunomodulatory proteins to interact with the host. In order to demonstrate their production in this species, EVs were purified from cell-free culture supernatants of the probiotic strain *P. freudenreichii* CIRM-BIA 129, and their physicochemical characterization, using transmission electron microscopy and nanoparticle tracking analysis (NTA), revealed shapes and sizes typical of EVs. Proteomic characterization showed that EVs contain a broad range of proteins, including immunomodulatory proteins such as SlpB. *In silico* protein-protein interaction predictions indicated that EV proteins could interact with host proteins, including the immunomodulatory transcription factor NF-κB. This potential interaction has a functional significance because EVs modulate inflammatory responses, as shown by IL-8 release and NF-κB activity, in HT-29 human intestinal epithelial cells. Indeed, EVs displayed an anti-inflammatory effect by modulating the NF-κB pathway; this was dependent on their concentration and on the proinflammatory inducer (LPS-specific). Moreover, while this anti-inflammatory effect partly depended on SlpB, it was not abolished by EV surface proteolysis, suggesting possible intracellular sites of action for EVs. This is the first report on identification of *P. freudenreichii-*derived EVs, alongside their physicochemical, biochemical and functional characterization. This study has enhanced our understanding of the mechanisms associated with the probiotic activity of *P. freudenreichii* and identified opportunities to employ bacterial-derived EVs for the development of bioactive products with therapeutic effects.

## Introduction

Intercellular communication is an essential biological process that involves several soluble biomolecules that may be secreted, surface-exposed or packed inside extracellular vesicles (EVs) (Gho and Lee, 2017; Toyofuku, 2019). EVs are lipid bilayer nanoparticles which range in size from 20 to 300 nm and are released by cells from all living kingdoms (Brown et al., 2015; Kim et al., 2015a; Liu et al., 2018a). They play a pivotal role in cell-to-cell communication through their ability to transport bioactive molecules (proteins, nucleic acids, lipids, metabolites) from donor to recipient cells. Bacterial EVs are implicated in virulence factor delivery, antibiotic resistance, competition, survival, and host cell modulation (Kim et al., 2015b; Toyofuku et al., 2019).

The participation of EVs in the beneficial roles of probiotic bacteria has been increasingly reported (Molina-Tijeras et al., 2019). The release of EVs by *Lactobacillus* species is well documented; *Lactobacillus reuteri* DSM 17938-derived EVs are associated with extracellular DNA-dependent biofilm formation (Grande et al., 2017) and EVs secreted by *Lactobacillus casei* BL23 have also been reported and shown to contain diverse biomolecules which include nucleic acids and proteins previously associated with its probiotic effects, such as p40 and p75 (Rubio et al., 2017). *Lactobacillus rhamnosus* GG-derived EVs have been associated with the apoptosis of hepG2 cancer cells (Behzadi et al., 2017), and *Lactobacillus plantarum* WCFS1-derived EVs modulated the response of human cells to vancomycin-resistant enterococci (Li et al., 2017). Moreover, EVs derived from other probiotic species, such as *Bifidobacterium longum* KACC 91563, impact host cell responses by inducing mast cell apoptosis, which has implications for the treatment of food allergies (Kim et al., 2016). Furthermore, probiotic strains of *Escherichia coli* release outer membrane vesicles (OMVs) that are involved in reinforcement of the gastrointestinal epithelial barrier (Alvarez et al., 2016), the regulation of inflammatory responses and intestinal homeostasis, via the NOD1-signaling pathway (Cañas et al., 2018).

*Propionibacterium freudenreichii* has also been regarded consistently as a probiotic species, mainly because of its immunomodulatory properties and protective effects against experimentally induced inflammation *in vivo* (Lan et al., 2007a, 2008; Foligne et al., 2010; Cousin et al., 2012; Rabah et al., 2018a; Do Carmo et al., 2019). *P. freudenreichii* is a Gram-positive, pleiomorphic, microaerophilic dairy bacterium that is generally recognized as safe (GRAS) and has a qualified presumption of safety (QPS) status (Loux et al., 2015; Deutsch et al., 2017; Rabah et al., 2017). This species is also known for its involvement in the ripening, texture, and flavor of cheese (Ojala et al., 2017) and in vitamin B12 synthesis (Deptula et al., 2017). A *P. freudenreichii* strain was recently isolated from the gut microbiota of a human breast milk-fed preterm infant, suggesting that this species could also be considered as a commensal inhabitant of the human digestive tract (Colliou et al., 2017).

As for the molecular mechanisms underlying its probiotic effects, some studies have focused on identifying the surface proteins of *P. freudenreichii* and their role in cytokine induction (Le Maréchal et al., 2015; Deutsch et al., 2017; Do Carmo et al., 2019). Notably, cell wall-related proteins, S-layer type proteins, moonlighting proteins and proteins related to interactions with the host have been identified as important actors in immunomodulation of *P*. *freudenreichii* strain CIRM-BIA 129 (Le Maréchal et al., 2015). Specifically, recent studies reported the role of surface-layer protein B (SlpB) from this strain in bacterial adhesion to intestinal HT-29 cells and immunomodulation (Do Carmo et al., 2017, 2019; Rabah et al., 2018b), as well as that of large surface layer protein A (LspA) from strain P. UF1 in the regulation of colonic dendritic cells during inflammation via SIGNR1 binding (Ge et al., 2020). As well as surface proteins, additional metabolites may contribute to the probiotic effect, such as 1,4-Dihydroxy-2-naphthoic acid (DHNA) from *P. freudenreichii* ET3, which is linked to AhR pathway activation (Fukumoto et al., 2014). DHNA has also been implicated in colitis regression (Okada et al., 2013). Moreover, short-chain fatty acids (SCFAs) from strains TL133 and TL142 have been demonstrated to play a role in inducing apoptosis of tumor cell lines (Lan et al., 2007b, 2008; Cousin et al., 2016).

In view of the fact that EVs are emerging as important carriers of biologically active cargos and that vesiculogenesis is a generally occurring phenomenon, we hypothesized that they might explain some of the probiotic properties of *P. freudenreichii.* For the first time, our findings have shown that this species produces EVs, and we have characterized their physiochemical, biochemical and functional features. We report that *P. freudenreichii* CIRM-BIA 129-derived EVs are implicated in its anti-inflammatory properties, via modulation of the NF-κB pathway, thus building on knowledge regarding this important probiotic bacterium.

## Materials and Methods

### Bacterial Strain and Growth Conditions

*Propionibacterium freudenreichii* CIRM-BIA 129 (equivalent to the ITG P20 strain) was supplied, stored and maintained by the CIRM-BIA Biological Resource Center (Centre International de Ressources Microbiennes-Bactéries d’Intérêt Alimentaire, INRAE, Rennes, France). *P. freudenreichii* CIRM-BIA 129 and its isogenic *P. freudenreichii* CIRM-BIA 129 Δ*slpB* mutant strain (Do Carmo et al., 2017) were cultured in cow milk ultrafiltrate (UF) supplemented with 100 mM sodium lactate and 5 g L^–1^ casein hydrolysate at 30°C and without agitation, until stationary phase (72 h of incubation, 2 × 10^9^ CFU mL^–1^), as reported previously (Cousin et al., 2012).

### Purification of EVs

Cells were pelleted by the centrifugation (6000 *g*, 15 min, room temperature) of cultures in UF (500 mL) and the supernatant fraction was filtered using 0.22 μm Nalgene top filters (Thermo Scientific) to remove any remaining bacterial cells. The supernatant was then concentrated 1000 times using Amicon ultrafiltration units with a 100 kDa cut-off point in successive centrifugations at 2500 *g*. The concentrated suspension of EVs was recovered in TBS buffer (Tris-Buffered Saline, 150 mM NaCl; 50 mM Tris–HCl, pH 7.5) and further purified by size exclusion chromatography (qEV original 70 nm; iZON), as recommended by the manufacturer (Böing et al., 2014). Briefly, 0.5 mL of EV samples was applied to the top of the chromatographic column, followed by TBS buffer for elution. Then, fractions of 0.5 mL were recovered in separate tubes. Fractions 1–6 were discarded as void, EVs-containing fractions (fractions 7–9) were pooled together and the remaining fractions were discarded due to protein contamination or low EV content.

### Negative Staining for Transmission Electron Microscopy

To characterize the shape of purified EVs, negative staining electron microscopy was conducted as previously described (Tartaglia et al., 2018). Briefly, a drop of EV solution was applied on a glow-discharged formvar-coated copper EM grid and blotted with a filter paper to remove excess solution. A drop of 2% uranyl acetate was applied to the EM grid, blotted again and finally dried before imaging under a Jeol 1400 transmission electron microscope (JEOL Ltd.) operating at 120 Kv.

### Nanoparticle Tracking Analysis for EV Size and Concentration Assessment

To measure the size and concentration of EVs, nanoparticle tracking analysis (NTA) was performed at 25.0°C using a NanoSight NS300 instrument (Malvern Panalytical) with a sCMOS camera and a Blue488 laser (Mehdiani et al., 2015). Samples were applied in constant flux with a syringe pump speed of 50. For each measurement, 5×60-s videos were recorded with camera level 15. Other parameters were adjusted accordingly to achieve image optimization.

### Proteomic Analysis

Three independent biological replicates of purified EVs from *P. freudenreichii* CIRM-BIA 129 (approximately 1 μg per sample) and the whole cell proteome were separated and visualized using 12% SDS-PAGE (Laemmli, 1970) and silver staining (Switzer et al., 1979). Next, EV proteins were hydrolyzed with trypsin for NanoLC-ESI-MS/MS analysis, as previously described (Gagnaire et al., 2015; Huang et al., 2016). Briefly, gel pieces were washed with acetonitrile and ammonium bicarbonate solution and dried under a vacuum. Next, in-gel trypsin digestion was performed overnight at 37°C and stopped with trifluoroacetic acid (Sigma-Aldrich). After digestion, the peptides were identified from the MS/MS spectra using X!TandemPipeline software (Langella et al., 2017) and searches were performed against the genome sequence of *P. freudenreichii* CIRM-BIA 129. The database search parameters were specified as follows: trypsin cleavage was used and the peptide mass tolerance was set at 10 ppm for MS and 0.05 Da for MS/MS. Methionine oxidation was selected as a variable modification. For each peptide identified, a maximum *e*-value of 0.05 was considered to be a prerequisite for validation. A minimum of two peptides per protein was imposed, resulting in a false discovery rate (FDR) of 0.15% for protein identification.

Proteomic data were further analyzed and visualized using Python libraries Pandas, NumPy, Matplotlib, and Seaborn. Functional annotations and Clusters of Orthologous Groups (COGs) were obtained using the eggNOG-mapper v2 web tool (Huerta-Cepas et al., 2017, 2019), while proteins and gene data were retrieved from NCBI and Uniprot (Bateman, 2019). Subcellular location prediction was performed with CELLO2GO (Yu et al., 2014) and the prediction of lipoproteins was conducted using PRED_LIPO (Bagos et al., 2008).

### Prediction of Protein-Protein Interactions

In order to screen for potential biological functions of EVs, the prediction of interactions between EV proteins and human proteins was carried out. The reference human proteome was retrieved from Uniprot (UP000005640) and contained 74,788 protein sequences. For the first method of prediction, EVs and human proteins were submitted to the InterSPPI web server (Lian et al., 2019), a machine-learning-based predictor. For the second method of prediction, a interolog-based approach was used (Folador et al., 2014), establishing homology relationships with the interactions described in the String and Intact databases (Kerrien et al., 2012; Szklarczyk et al., 2017). The resulting interactions were filtered according to the prediction scores (intersppi: minimum score of 0.9765, for a specificity 0.99; interolog: minimum score of 500 out of 1000). Next, the dataset was reduced to a canonical representation, only retaining the human protein isoform appearing in highest-scoring interactions and removing non-reviewed human proteins. For the predicted interactions, the human counterpart was programmatically mapped to KEGG pathways in order to identify the associated functional modules. Data analysis and graphic representations were obtained using Python libraries Pandas, Seaborn, Matplotlib, Matplotlib_venn, and Cytoscape software (Shannon, 2003).

### Culture of Eukaryotic Cells

HT-29 human epithelial cells were used for immunomodulation assays; either the parental lineage (HT-29, colon adenocarcinoma; ATCC HTB-38) or a lineage transfected with the secreted alkaline phosphatase (SEAP) reporter gene for NF-kB activation monitoring (HT-29/kb-seap-25) (Lakhdari et al., 2010). The reporter HT-29/kb-seap-25 cells were cultured in RPMI-Glutamine medium (Sigma-Aldrich), supplemented with 10% fetal bovine serum (Corning), 1% non-essential amino acids, 1% sodium pyruvate, 1% HEPES buffer (Thermofisher Scientific), and 1% penicillin-streptomycin (Lonza) according to Lakhdari et al. (2010). The parental HT-29 cells were cultured in high-glucose DMEM medium (Dominique Dutscher) supplemented with 10% fetal bovine serum and 1% penicillin-streptomycin (Do Carmo et al., 2017). For subcultures, cells were rinsed with DPBS (Thermofisher Scientific) and detached with a trypsin (0.05%) – EDTA (0.02%) solution (Sigma). Periodically, 100 μg/mL Zeocin (Invivogen) was applied to the HT-29/kb-seap-25 cell culture in order to maintain selective pressure on the cells containing the transfected plasmid.

### NF-κB Modulation Assays

HT-29/kb-seap-25 cells were seeded on 96-well plates at 3 × 10^4^ cells/well and incubated for 24 h at 37°C under 5% CO_2_ prior to stimulation (Lakhdari et al., 2010). Monolayer confluence was checked under the microscope before and after every stimulation. TNFα (1 ng mL^–1^; PeproTech), IL-1β (1 ng mL^–1^; Invivogen), and LPS from *E. coli* O111:B4 (1 ng mL^–1^; L3024-5MG, Sigma-Aldrich) were used to induce inflammation. The cells were stimulated with the samples (controls and EV preparations) and inflammation inducers for 24 h. The supernatants from all the wells were then revealed with Quanti-Blue^TM^ reagent (Invivogen) to assess SEAP activity. Cell proliferation was evaluated under all conditions using the CellTiter 96^®^ AQueous One Solution Cell Proliferation Assay (MTS, Promega), according to the manufacturer’s instructions. Absorbance was read at 655 nm for the SEAP activity assay and at 490 nm for the MTS assay using a Xenius (SAFAS Monaco) microplate reader. For surface protein assays, 10^9^ EV ml^–1^ were applied directly or after incubation at 37°C for 1 h in the presence or absence of proteinase K (20 μg mL^–1^; Qiagen), in order to evaluate a possible role for EV surface proteins in immunomodulation.

### ELISA Cytokine Assay

Enzyme-linked immunosorbent assay (ELISA) tests tests were performed under the same conditions as the NF-κB modulation assays using HT-29 parental cells. The human IL-8/CXCL8 DuoSet (R&D Systems) kit was used to evaluate cell culture supernatants after stimulation, according to the manufacturer’s instructions. Absorbance was read at 450 nm using a Xenius (SAFAS Monaco) microplate reader.

### Statistical Analysis

All experiments were conducted independently and in triplicate at least, and the results are expressed as means ± standard deviations of biological replicates. For absorbance measurements, the values were normalized by the control condition. The differences between groups were verified using one-way ANOVA followed by Tukey’s multiple comparisons test with GraphPad Prism (GraphPad Software, San Diego, CA, United States).

## Results

### *Propionibacterium freudenreichii* Produces Extracellular Vesicles

**FIGURE 1:**
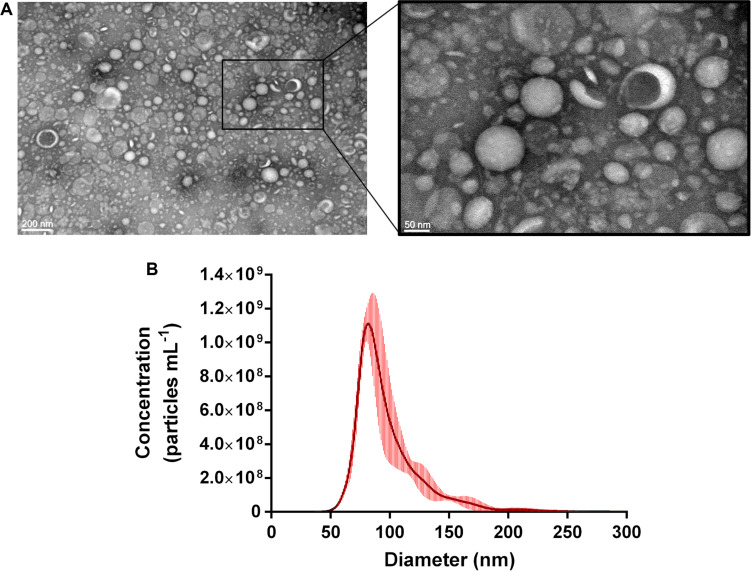
*P. freudenreichii* CIRM-BIA 129 secretes extracellular vesicles. **(A)** Transmission electron microscopy image after the negative staining of *P. freudenreichii*-secreted EVs purified from UF culture supernatants. **(B)** Size distribution (diameter) of purified EVs as measured by nanoparticle tracking analysis (NTA). Concentration data are expressed as mean ± standard deviation from three independent biological replicates.

In order to determine whether *P. freudenreichii* produced EVs, strain CIRM-BIA 129 was cultured in cow milk UF medium and EVs were purified from the cell-free supernatants of stationary phase cultures. As a control, we checked that EVs were absent from the UF medium before being used for bacterial culture. Visualization by electron microscopy revealed that *P. freudenreichii* strain CIRM-BIA 129 produced EVs of a typical shape, i.e., spherical cup-shaped structures (Figure 1A). Size characterization by NTA showed that the EVs presented a monodisperse profile with modal diameter of 84.80 ± 2.34 nm (Figure 1B).

### *P. freudenreichii-*Secreted EVs Contain a Functionally Diverse Set of Proteins, Including Immunomodulatory Proteins

**FIGURE 2:**
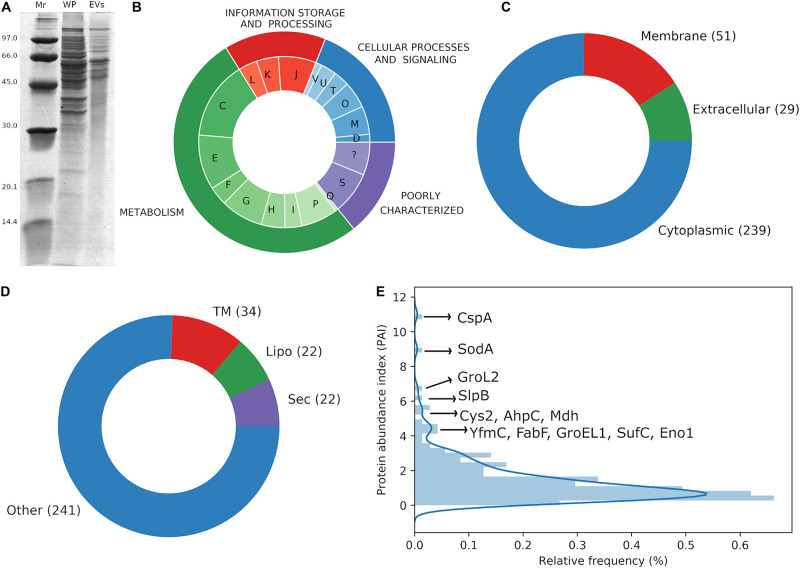
*P. freudenreichii*-secreted EVs contain proteins involved in interactions with the host. **(A)** SDS-PAGE showing the protein profile of the whole proteome (WP) and vesicular proteome (EVs). Molecular weight standards are indicated on the left (Mr, kDa). **(B)** Functional annotation of EV proteins by the prediction of COG categories: D – Cell cycle control, cell division, chromosome partitioning, M – Cell wall/membrane/envelope biogenesis, O – Post-translational modification, protein turnover, and chaperones, T – Signal transduction mechanisms, U – Intracellular trafficking, secretion, and vesicular transport, V – Defense mechanisms, J – Translation, ribosomal structure and biogenesis, K – Transcription, L – Replication, recombination and repair, C – Energy production and conversion, E – Amino acid transport and metabolism, F – Nucleotide transport and metabolism, G – Carbohydrate transport and metabolism, H – Coenzyme transport and metabolism, I – Lipid transport and metabolism, P – Inorganic ion transport and metabolism, Q – Secondary metabolites biosynthesis, transport, and catabolism, S – Function unknown, ? – Not predicted. **(C)** Subcellular localization of EV proteins as predicted by Cello2GO. **(D)** Prediction of lipoproteins by PRED-LIPO: Sec: secretion signal peptide, Lipo: lipoprotein signal peptide, TM: transmembrane, Other: no signals found. **(E)** Protein abundance index (PAI) frequency distribution for EV proteins, highlighting the most frequently expressed proteins.

Cargo proteins associated with *P. freudenreichii*-secreted EVs were determined by Nano LC-ESI-MS/MS analysis from three biological replicates of EVs. A total of 319 proteins was identified consistently in EVs derived from UF medium cultures (Supplementary Table S1), which corresponds to 11% of the whole theoretical proteome of *P. freudenreichii* CIRM-BIA 129. Figure 2A confirmed a much more complex proteome in the whole cell *P. freudenreichii* extract than in the EV extract.

The proteins associated with these EVs were distributed between most of the COG categories (Figure 2B). The majority of proteins could be assigned to COGs related to the general category of “metabolism,” e.g., energy production and conversion (C, 14.8%), amino acid transport and metabolism (E, 10.4%), and carbohydrate transport and metabolism (G, 8%). The most common COGs in the general category of “information, storage and processing” were translation, ribosomal structure and biogenesis (J, 7.1%) and transcription (K, 4.2%). Finally, cell wall/membrane/envelope biogenesis (M, 5.3%) and post-translational modifications, protein turnover and chaperones (O, 5%), were the most frequently counted COGs in the general category of “cellular processes and signaling.” Interestingly, several proteins previously identified as important actors in immunomodulation were packed within EVs: enolase (Eno1, PFCIRM129_06070), aconitase (Acn, PFCIRM129_04640), glutamine synthetase (GlnA1, PFCIRM129_11730), glucose-6-phosphate isomerase (Gpi, PFCIRM129_10645), triosephosphate isomerase (Tpi1, PFCIRM129_11290), the surface-layer proteins SlpB (PFCIRM129_00700) and SlpE (PFCIRM129_05460), the BopA solute binding protein (PFCIRM129_08120), internaline A (InlA, PFCIRM129_12235), the hypothetical protein PFCIRM129_10785 and the GroL2 chaperonin (PFCIRM129_10100) (Le Maréchal et al., 2015; Deutsch et al., 2017; Do Carmo et al., 2017).

Regarding predictions of the subcellular localization of the proteins, they were mainly predicted to be cytoplasmic (*n* = 239), but some membrane (*n* = 51), and extracellular (*n* = 29) proteins were also identified (Figure 2C). Lipoprotein signal peptides were also predicted in a small fraction of the proteins (*n* = 22). Other proteins were predicted to contain a secretory signal peptide I (*n* = 22) and transmembrane motifs (*n* = 34) (Figure 2D). Regarding the protein abundance index (Ishihama et al., 2005), it displayed a non-normal distribution with a tail of highly expressed proteins, including immunomodulatory SlpB, 60 kDa chaperonin 2 (GroL2) and enolase 1 (Eno1), as well as cold shock-like protein CspA, iron/manganese superoxide dismutase (SodA), cysteine synthase 2 (Cys2), alkyl hydroperoxide reductase subunit C (AhpC), and malate dehydrogenase (Mdh) (Figure 2E).

### The Proteins From *P. freudenreichii*-Secreted EVs Potentially Interact With Human Immunomodulatory Proteins

**FIGURE 3:**
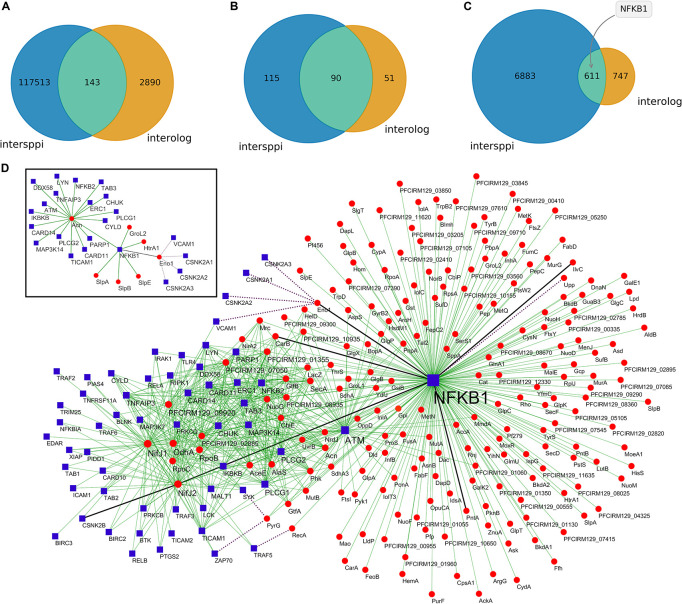
Predictions of protein-protein interactions suggest a modulation of the NF-κB signaling pathway by *P. freudenreichii*-secreted EVs. **(A)** Number of interactions predicted by each methodology. **(B)** Number of bacterial proteins predicted to interact as a function of the prediction method. **(C)** Number of human proteins predicted to interact as a function of the prediction method, highlighting the top-interacting NFKB1 protein. **(D)** Subnetwork of predicted interactions mapping to the KEGG NF-κB pathway. Blue square nodes represent human proteins, red round nodes represent bacterial proteins, full thin green lines represent intersppi-predicted interactions, dotted purple lines represent interolog-predicted interactions and full thick black lines represent interactions shared by both methods. Inset: selected interactions involving bacterial proteins previously associated with immunomodulatory roles.

The proteins found in *P. freudenreichii*-derived EVs were tested against the human proteome *in silico* in order to predict their interactions. Machine learning-based (intersppi) and homology-based (interolog) methods were employed for this task. There was a considerable difference regarding the number of predicted interactions which depended on the method used. Intersppi predicted 117,513 interactions, while the interolog method predicted 2,890 interactions; there were 143 common interactions between the two methods (Figure 3A and Supplementary Tables S2–S4). Regarding interacting bacterial proteins, 115 proteins appeared exclusively in intersppi interactions, 51 proteins appeared exclusively in interolog interactions and 90 proteins appeared in interactions predicted by both methods (Figure 3B and Supplementary Table S5). As for interacting human proteins, the majority was predicted by the intersppi method only (6,883 proteins), whereas 747 proteins appeared exclusively in interolog interactions and 611 proteins appeared in interactions shared by both methods of prediction (Figure 3C and Supplementary Table S6).

The predicted interactions mapped to diverse KEGG terms, including metabolism, signal transduction, infectious diseases and the immune system (Supplementary Figure S1). Interestingly, the nuclear factor NF-κB p105 subunit (NFKB1, P19838) was the most frequent interacting human protein considering common and intersppi-exclusive interactions. The subnetwork of interactions mapping to the KEGG NF-κB signaling pathway (Figure 3D) included both interactions with adapter molecules (e.g., TICAM1, TICAM2, TRAF6) and Toll-like receptors (e.g., TLR1, TLR4, TLR5, TL6), as well as NFKB1 itself (Supplementary Figures S2, S3). These results therefore indicated some interesting potential roles for EV proteins in immunomodulation that need to be verified experimentally.

### *P. freudenreichii*-Secreted EVs Modulate the NF-κB Pathway in a Dose and Inducer-Dependent Manner

In view of the finding that *in silico* predictions showed interactions between EV proteins and human immunomodulatory proteins (and particularly NFKB1), together with previous evidence of immunomodulatory roles for *P. freudenreichii*, we investigated this potential *in vitro*. A cellular reporter system regarding modulation of the regulatory activity of the NF-κB transcription factor (HT-29/kb-seap-25) was therefore employed (Lakhdari et al., 2010). Cells were kept in contact with proinflammatory inducers (LPS, TNF-α or IL-1β) and EV preparations, in order to test the ability of EVs to attenuate the induced inflammatory response.

**FIGURE 4:**
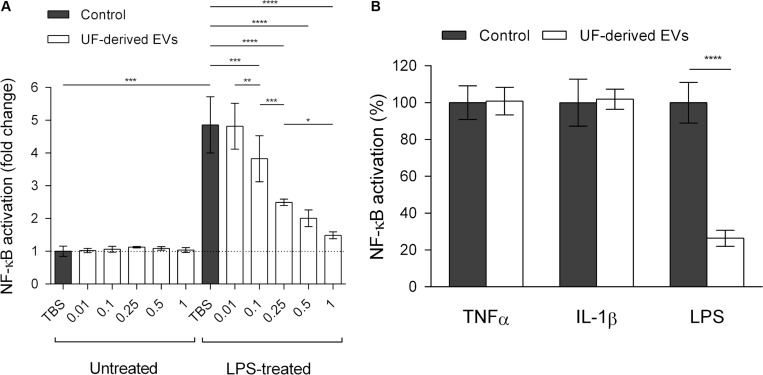
*P. freudenreichii*-secreted EVs specifically mitigate LPS-induced NF-κB activation in intestinal epithelial cells. **(A)** Measure of regulatory activity of the NF-κB transcription factor in HT-29 intestinal epithelial cells untreated or treated with LPS (1 ng mL^–1^) in the presence of TBS buffer control or different concentrations of EVs purified from the supernatants of *P. freudenreichii* CIRM-BIA 129 culture in UF medium. EV concentrations: 0.01 = 1.0 × 10^7^ EVs ml^–1^, 0.1 = 1.0 × 10^8^ EVs ml^–1^, 0.25 = 2.5 × 10^8^ EVs ml^–1^, 0.5 = 5.0 × 10^8^ EVs ml^–1^, 1 = 1.0 × 10^9^ EVs ml^–1^. **(B)** Percentage NF-κB activation in HT-29 intestinal epithelial cells after stimulation by LPS (1 ng mL^–1^), TNF-α (1 ng mL^–1^) or IL-1β (1 ng mL^–1^) inducers, in the presence or absence of UF-derived EVs (1.0 × 10^9^ EVs ml^–1^). The values are normalized by the control conditions (stimulation by the inducer in the absence of EVs). ANOVA with the Tukey’s multiple comparison test: *P < 0.05, **P < 0.01, ***P < 0.001, ****P < 0.0001.

Among cells that were not treated with proinflammatory inducers, their exposure to EVs did not affect the activity of the reporter, keeping a basal level of NF-κB activity (Figure 4A). When HT-29/kb-seap-25 cells were treated with LPS in the absence of EVs, NF-κB activation increased, thus showing LPS-dependent induction of the NF-κB pathway. With the addition of EVs at increasing concentrations, a dose-dependent reduction of NF-κB activation was observed. With the highest EV concentration tested (1.0 × 10^9^ EVs ml^–1^), NF-κB activation was comparable to that of untreated control cells. Furthermore, when the EV concentration was kept at a constant level, NF-κB modulation was also dependent on the pathway inducer. In the presence of EVs, there was a significant reduction in NF-κB activation in LPS-treated cells, but not in cells treated with other inducers (TNF-α and IL-1β) (Figure 4B).

### *P. freudenreichii*-Secreted EVs Also Modulate IL-8 Release in a Dose and Inducer-Dependent Manner

To further investigate the anti-inflammatory role suggested by the reduction in NF-κB activity, release of the proinflammatory chemokine IL-8 by HT-29 cells was determined in the presence of various EV concentrations and proinflammatory inducers.

**FIGURE 5:**
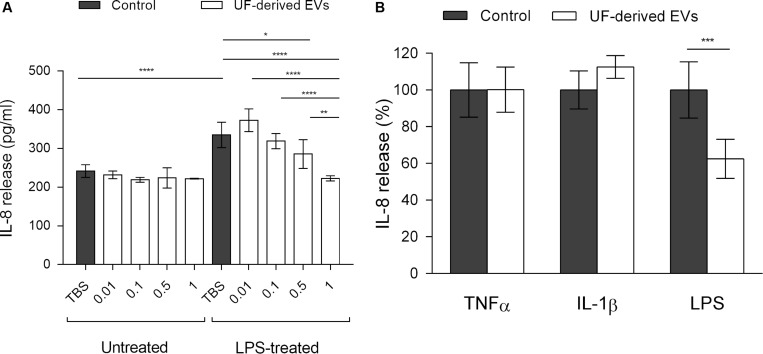
*P. freudenreichii*-secreted EVs mitigate the release of IL-8 induced by LPS. **(A)** Concentration of IL-8 (pg ml^–1^) released by HT-29 intestinal epithelial cells untreated or treated with LPS (1 ng mL^–1^) in the presence of different concentrations of EVs purified from the supernatants of *P. freudenreichii* CIRM-BIA 129 culture in UF medium. EV concentrations: 0.01 = 1.0 × 10^7^ EVs ml^–1^, 0.1 = 1.0 × 10^8^ EVs ml^–1^, 0.5 = 5.0 × 10^8^ EVs ml^–1^, 1 = 1.0 × 10^9^ EVs ml^–1^. **(B)** Percentage of IL-8 released by HT-29 intestinal epithelial cells after stimulation by LPS (1 ng mL^–1^), TNF-α (1 ng mL^–1^) or IL-1β (1 ng mL^–1^) inducers, in the presence or absence of UF-derived EVs (1.0 × 10^9^ EVs ml^–1^). The values are normalized by the control conditions (stimulation by inducer in the absence of EVs). One-way ANOVA followed by Tukey’s (left panel) or Dunnett’s (right panel) multiple comparisons test: *P < 0.05, **P < 0.01, ***P < 0.001, ****P < 0.0001.

In the absence of proinflammatory inducers, EVs had no effect on IL-8 release from HT-29 cells (Figure 5A). In the TBS buffer control group, LPS-treated HT-29 cells displayed an increase in IL-8 release when compared to untreated cells, reflecting the LPS-induced proinflammatory effect. In the presence of EVs, a significant and dose-dependent decrease of IL-8 release by LPS-stimulated cells was observed. At the highest concentration (1.0 × 10^9^ EVs ml^–1^), IL-8 release was reduced down to a level comparable to that seen in untreated control cells.

Moreover, as in the case of NF-κB activity, the EV-mediated reduction in IL-8 release was specific to treatment with the LPS proinflammatory inducer (Figure 5B). When cells were treated with TNF-α or IL-1β, no effect of EVs could be detected on IL-8 release by HT-29 cells. Taken together, these results showed that *P. freudenreichii*-derived EVs were endowed with anti-inflammatory properties that depended on the concentration and inflammatory stimulus.

### *P. freudenreichii*-Secreted EVs Are Not Cytotoxic Against Intestinal Epithelial Cells

**FIGURE 6:**
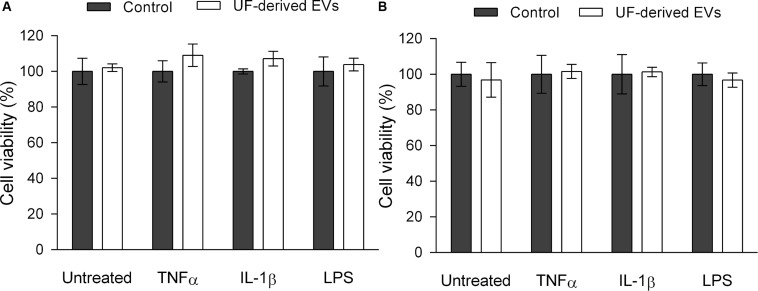
*P. freudenreichii*-secreted EVs are not cytotoxic against intestinal epithelial cells. Viability percentage of HT-29 intestinal epithelial cells **(A)** and HT-29/kb-seap-25 cellular reporter cell line **(B)** before or after stimulation by LPS (1 ng mL^–1^), TNF-α (1 ng mL^–1^) or IL-1β (1 ng mL^–1^) inducers, in the presence or absence of UF-derived EVs (1.0 × 10^9^ EVs ml^–1^). The values are normalized by the control conditions (control buffer or stimulation by an inducer in the absence of EVs). There are no statistically significant differences between group means as determined by one-way ANOVA followed by Tukey’s multiple comparisons test.

In order to ensure that reductions in NF-κB activation and IL-8 release were associated with regulatory activity and not simply with cell death, an MTS cell proliferation assay was also performed (Figure 6). This assay showed that there was no significant difference in cell viability between the control and test groups in terms of both parental HT-29 and HT-29/kb-seap-25 reporter cells.

### Influence of Surface Proteins on NF-κB Modulation

**FIGURE 7:**
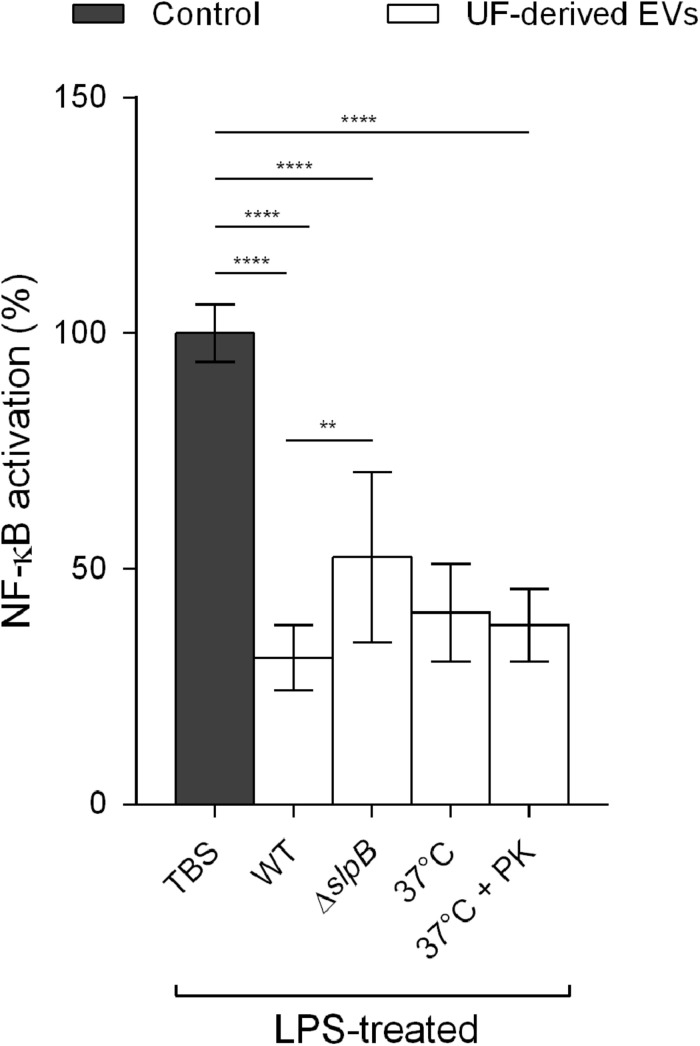
SlpB is required to achieve the effective mitigation of NF-κB activation in human intestinal epithelial cells. Percentage of NF-κB activation in HT-29 intestinal epithelial cells after stimulation by LPS (1 ng μL^–1^) in the presence or absence of UF-derived EVs (1.0 × 10^9^ EVs ml^–1^). WT: wild type *P. freudenreichii* CIRM-BIA 129-derived EVs, Δ*SlpB*: isogenic mutant *P. freudenreichii* CIRM-BIA 129 Δ*SlpB*-derived EVs, 37°C: wild type *P. freudenreichii* CIRM-BIA 129-derived EVs incubated at 37°C for 1 h, 37°C + PK: wild type *P. freudenreichii* CIRM-BIA 129-derived EVs incubated at 37°C for 1 h in the presence of proteinase K. The values are normalized by the control conditions (stimulation by an inducer in the absence of EVs). One-way ANOVA followed by Tukey’s multiple comparisons test: **P < 0.01, ****P < 0.0001.

Further tests were performed to determine whether the modulation of NF-κB by *P. freudenreichii* EVs was influenced by a surface-layer protein (SlpB), recognized as being immunomodulatory in the studied strain (Deutsch et al., 2017; Do Carmo et al., 2017; Figure 7). EVs derived from an isogenic mutant *P. freudenreichii* CIRM-BIA 129 Δ*slpB* and proven not to produce this specific protein, displayed a partial reduction of NF-κB activation, when compared to wild type-derived EVs. This suggested that SlpB plays a fundamental role in EV modulation of the NF-κB pathway, but it is likely that other important effectors also need to be considered.

Furthermore, in order to determine whether NF-κB pathway modulation by EVs is dependent on proteins that might be exposed on the EV surface, they were treated with proteinase K prior to the cellular assays (Figure 7). The incubation of EVs at 37°C for 1 h, in the presence or absence of proteinase K, did not result in significant differences in NF-κB activation, when compared to the wild-type untreated control. This suggested that these immunomodulatory effectors may be inside EVs and not at their surface.

## Discussion

Although research on bacterial EVs focused initially on Gram-negative and pathogenic bacteria, a major increase in the number of studies involving Gram-positive and probiotic bacteria has been seen during the past decade (Liu et al., 2018b; Molina-Tijeras et al., 2019). That is in line with the growing recognition of the widespread occurrence and diverse functions of EVs (Brown et al., 2015; Liu et al., 2018a, b). In the case of *P. freudenreichii*, a Gram-positive dairy probiotic bacterium, a recent study indicated the presence of an extracellular structure resembling a potential EV, which bulged out from the membrane of *P. freudenreichii* strain JS22 (Frohnmeyer et al., 2018), suggesting that this species might produce EVs. Nevertheless, ours is the first complete report to have identified the occurrence of EVs in *P. freudenreichii* strain CIRM-BIA 129, and included their physicochemical, biochemical and functional characterization. The EVs thus identified displayed the basic features of extracellular prokaryotic membrane vesicles, i.e., a nanometric size range, a cup-shaped morphology and a spherical structure (Raposo and Stoorvogel, 2013; Liu et al., 2018b; Tartaglia et al., 2018).

CIRM-BIA 129 EVs carry a diverse set of proteins that represent a broad range of biological functions. More than half of these proteins are related to metabolism, so that, initially, they may not appear to exert specific functions outside bacterial cells. However, the transport of metabolism-related proteins by EVs may represent a mechanism of functional exchange and complementation in the context of bacterial communities. For example, members of the *Bacteroides* genus share enzymes with the microbiome through OMVs, so as to contribute to the degradation of complex polysaccharides (Elhenawy et al., 2014; Rakoff-Nahoum et al., 2014; Lynch and Alegado, 2017). On the other hand, some of these metabolism-related proteins, from either *P. freudenreichii* CIRM-BIA 129, or from other strains in the species, have been implicated in interactions with the host. This is the case of enolase (Eno1, PFCIRM129_06070) and aconitase (Acn, PFCIRM129_04640) (Deutsch et al., 2017). Others, such as glutamine synthetase (GlnA1, PFCIRM129_11730), glucose-6-phosphate isomerase (Gpi, PFCIRM129_10645) and triosephosphate isomerase (Tpi1, PFCIRM129_11290), have also been described as moonlighting proteins, with adhesin functions, in other species (Kainulainen et al., 2012; Rodríguez-Bolaños and Perez-Montfort, 2019). As well as metabolism-related proteins, other proteins packed into EVs are also related to interactions between *P. freudenreichii* and the host: SlpB (PFCIRM129_00700) and SlpE (PFCIRM129_05460) surface-layer proteins, the BopA solute binding protein (PFCIRM129_08120), internaline A (InlA, PFCIRM129_12235), the hypothetical protein PFCIRM129_10785 and the GroL2 chaperonin (PFCIRM129_10100) (Le Maréchal et al., 2015; Deutsch et al., 2017; Do Carmo et al., 2017). It has been shown that SlpB mediates the adhesion of *P. freudenreichii* CIRM-BIA 129 to intestinal epithelial HT29 cells (Do Carmo et al., 2017), reduces LPS-induced IL-8 expression in HT-29 cells (Do Carmo et al., 2019) and participates in the induction of anti-inflammatory cytokines such as IL-10 in human peripheral blood mononuclear cells, mesenteric lymph nodes cells and epithelial HT29 cells (Foligne et al., 2010; Le Maréchal et al., 2015; Deutsch et al., 2017; Rabah et al., 2018a). Also, inactivation of the gene encoding SlpE suppresses IL-10 induction by *P. freudenreichii* CIRM-BIA 129 (Deutsch et al., 2017). It is interesting to note that SlpB, Eno1 and GroL2 were found to be some of the most abundant EV proteins, which may be a further indication of the potentially beneficial roles exerted by CIRM-BIA 129 EVs on host cells. This is in accordance with the potential immunomodulatory role suggested for these proteins in a multi-strain and multi-omics study (Deutsch et al., 2017).

In order to investigate potential roles for EVs in the context of host-microorganism interactions, we conducted *in silico* predictions of interactions between the bacterial proteins identified in EVs and human proteins. We employed a machine learning-based method (intersppi), which relies on protein sequences and network properties, to identify patterns of classification into groups of interacting or non-interacting protein pairs (Lian et al., 2019). We also used a homology-based prediction of interacting pairs (interolog) that relies on the mapping of similarities to interaction databases and interaction transfers among homologs (Folador et al., 2014). Our results revealed a considerable difference between the methods in the number and nature of the interactions predicted. This difference was mainly due to the fact that the interolog methodology depends on data availability in interaction databases, such as STRING and INTACT, from which homology mapping is performed (Wang et al., 2012). The data may therefore have been biased by experimental work focused on specific aspects and organisms, as well as the conservation of proteins, which may affect homology identification. For example, metabolism-related proteins tend to be more conserved, so they are therefore overrepresented in interactions predicted using the interolog methodology. On the other hand, intersppi aims to capture more general patterns of protein binding, enabling *ab initio* predictions that are not reliant on the availability of prior data. However, it is still susceptible to training data bias and a certain probability of false positives (Keskin et al., 2016).

The predicted interactions mapped to several immunology-related KEGG terms, such as signal transduction, infectious diseases and the immune system, thus shedding light on a possible immunomodulatory role for *P. freudenreichii* EVs. These predictions corroborate some previous findings which associated the *P. freudenreichii* bacterium with immunomodulatory roles (Foligne et al., 2010; Deutsch et al., 2017; Do Carmo et al., 2017; Frohnmeyer et al., 2018; Rabah et al., 2018a). Interestingly, the predicted data also suggested that this immunomodulation could involve the NF-κB pathway, since the nuclear factor NF-κB p105 subunit (NFKB1, P19838) was the most frequent interacting human protein according to the intersppi predictions and also the interactions shared between the two methods. Regarding bacterial proteins previously reported as being immunomodulatory, Acn, Eno1, GroL2, HtrA1, SlpA, SlpB, and SlpE were predicted to interact directly with NFKB1 using the intersppi method. In addition, the interaction between Eno1 and NFKB1 was also predicted by interolog. Other proteins in the NF-κB pathway were also predicted to interact with Acn: the inhibitor of nuclear factor kappa-B kinase subunits alpha (IKKA) and beta (IKKB), as well as TIR domain-containing adapter molecule 1 (TICAM1). Moreover, interactions with toll-like receptors (e.g., TLR1, TLR4, TLR5, TL6) suggested that the immunomodulatory role of EVs might also occur at the receptor level. Briefly, PPI data (i.e., highly interacting NFKB1 protein, interactions involving bacterial proteins previously demonstrated to be immunomodulatory and interactions mapping to KEGG terms related to the immune response) suggested an ability of EVs produced by *P. freudenreichii* CIRM-BIA 129 to exert an immunomodulatory effect via the NF-κB pathway, which then needed to be confirmed *in vitro*.

When tested on HT-29 intestinal epithelial cells, we found that EVs exert an inhibitory effect on LPS-induced IL-8 secretion, as was previously observed with intact *P. freudenreichii* CIRM-BIA 129 cells (Do Carmo et al., 2019). Their effect was dose-dependent and unrelated to side effects of EVs on cell viability. This immunomodulatory response was mediated through modulation of the regulatory activity of the NF-κB transcription factor. The NF-κB pathway leads to the upregulation of proinflammatory genes, being targeted by diverse pathogens and probiotic bacteria (Hayden and Ghosh, 2012; Mitchell et al., 2016). Therefore, some probiotics can downregulate the production of these proinflammatory cytokines, acting at different steps along the NF-κB pathway: *L. rhamnosus* GG and *Lactobacillus delbrueckii* subsp. *bulgaricus* downregulated p38 and IkB expression, respectively (Giahi et al., 2012), *L. plantarum* LM1004 and *Lactobacillus casei* DN-114 001 modulated the nuclear translocation of NF-κB (Tien et al., 2006; Lee et al., 2019), *Bacteroides thetaiotaomicron* promoted the nuclear export of RelA (Kelly et al., 2004), VSL#3 inhibited proteasome degrading activity (Petrof et al., 2004), and *Bifidobacterium breve* C50 decreased the phosphorylation of p38-MAPK and IkappaB-alpha (Heuvelin et al., 2009). Probiotic strains were also shown to exhibit immunoregulatory effects via the modulation of TLR negative regulators of the NF-κB pathway (Lakhdari et al., 2011; Kanmani and Kim, 2019). Specifically, *Lactobacillus helveticus* SBT2171 was reported to inhibit NF-κB activation by inducing A20 expression via TLR2 signal in LPS-stimulated peritoneal macrophages (Kawano et al., 2019). *Lactobacillus acidophilus* was shown to regulate the inflammatory response induced by enterotoxigenic *E. coli* K88 in piglets, notably through the increased expression of Tollip, IRAK-M, A20, and Bcl-3 (Li et al., 2016). *Lactobacillus paracasei* was associated to the inhibition of pro-inflammatory cytokines production by monocyte-macrophages, via the induction of A20, SOCS1, SOCS3, and IRAK3 (Sun et al., 2017).

The precise mechanism by which EVs produced by *P. freudenreichii* CIRM-BIA 129 modulate NF-κB activity still needs to be elucidated, but our work has provided some clues. EVs do not exert an immunomodulatory effect when the NF-κB pathway is stimulated by other inducers (such as TNF-α and IL-1β), indicating that EVs mitigate the activation of NF-κB via the LPS signaling pathway. These different ligands bind to specific receptors at the cell surface in order to activate the NF-κB pathway. Once bound, they use the same signal transduction mechanisms to activate the pathway. Therefore, EVs probably act at a level of the NF-κB pathway that is not common to the three inducers: TLR4, CD14, LBP, MD-2 and the TICAM1 and TICAM2 TIR domain-containing adaptor proteins. It is interesting to note that *in silico* prediction of protein-protein interactions predicted interactions with TLR4, the cell-surface receptor for LPS, but not with the TNF-α and IL-1β receptors. Furthermore, proteinase-treated EVs conserved their immunomodulatory properties. Therefore, the EV-triggered inhibition of NF-κB does not signal through binding between EV surface exposed proteins and the LPS receptor (i.e., LBP, TLR-4, CD14, MD-2). One can suppose that non-proteinaceous inhibitors may also be involved. However, to date, the immunomodulatory properties of *P. freudenreichii* have been associated with proteins. Moreover, EVs produced by a Δ*slpB P. freudenreichii* mutant partly lost their immunomodulation effects, indicating that several proteins, including SlpB, play a direct or indirect role in the anti-inflammatory response mediated by EVs. These results also suggest that these immunomodulatory proteins are packed into EVs and are not surface exposed. It cannot be excluded that immunomodulatory proteins interact directly with LPS receptors after their release from the lysis of EVs in the vicinity of cells, but it does seem more likely that they target specific intercellular components of the LPS-induced NF-κB pathway after EV uptake by membrane fusion or endocytosis (Mulcahy et al., 2014; Jefferies and Khalid, 2020).

It is now well recognized that EVs act as proxies of their parental cells. Accordingly, *P. freudenreichii* and its EVs share common features, notably their immunomodulatory effects mediated by SlpB. Whether *P. freudenreichii* also signals through the same pathway as its EVs constitutes a basis for further studies. Likewise, whether *P. freudenreichii* EVs exert immunomodulatory effects *in vivo*, as has been shown for the bacterium (Foligne et al., 2010; Rabah et al., 2018a; Do Carmo et al., 2019) is a challenging question that should be addressed in future research and with respect to potential EV-based probiotic applications. In sum, this study reflects efforts to demonstrate the widespread occurrence and functional diversity of EVs, particularly in a group of emerging EVs research, such as Gram-positive probiotic bacteria. It also contributes to a clearer understanding of the mechanisms associated with the probiotic traits of *P. freudenreichii* while opening up possibilities of employing bacterial-derived EVs for functional cargo delivery and for the development of novel probiotic products.

## Data Availability Statement

The datasets generated for this study can be found here: https://data.inrae.fr/dataset.xhtml?persistentId=doi:10.15454/Q6PPXY.

## Author Contributions

VR, GJ, YL, VC, and EG conceived and designed the experiments. VR, VB-B, JJ, BL, and HR performed the experiments. VR, EF, AN, JJ, and EG analyzed the data. FC, AN, JJ, VB-B, HB, NL, GJ, and EG gave practical suggestions to perform experiments. VC, YL, and EG contributed to funding acquisition. VR and EG wrote the original draft. All authors contributed to data interpretation, drafting the manuscript, critically revising the manuscript, and approving its final version.

## Conflict of Interest

The authors declare that the research was conducted in the absence of any commercial or financial relationships that could be construed as a potential conflict of interest.
