# Supplementary material for: Extracellular Vesicles Produced by the Probiotic Propionibacterium freudenreichii CIRM-BIA 129 Mitigate Inflammation by Modulating the NF-κB Pathway
Source: Front Microbiol. 2020 Jul 7;11:1544. doi: 10.3389/fmicb.2020.01544 (PMC7359729; doi:10.3389/fmicb.2020.01544)
Supplement: Supplementary file 2 [file Table_2.DOCX]

Supplementary Material

**
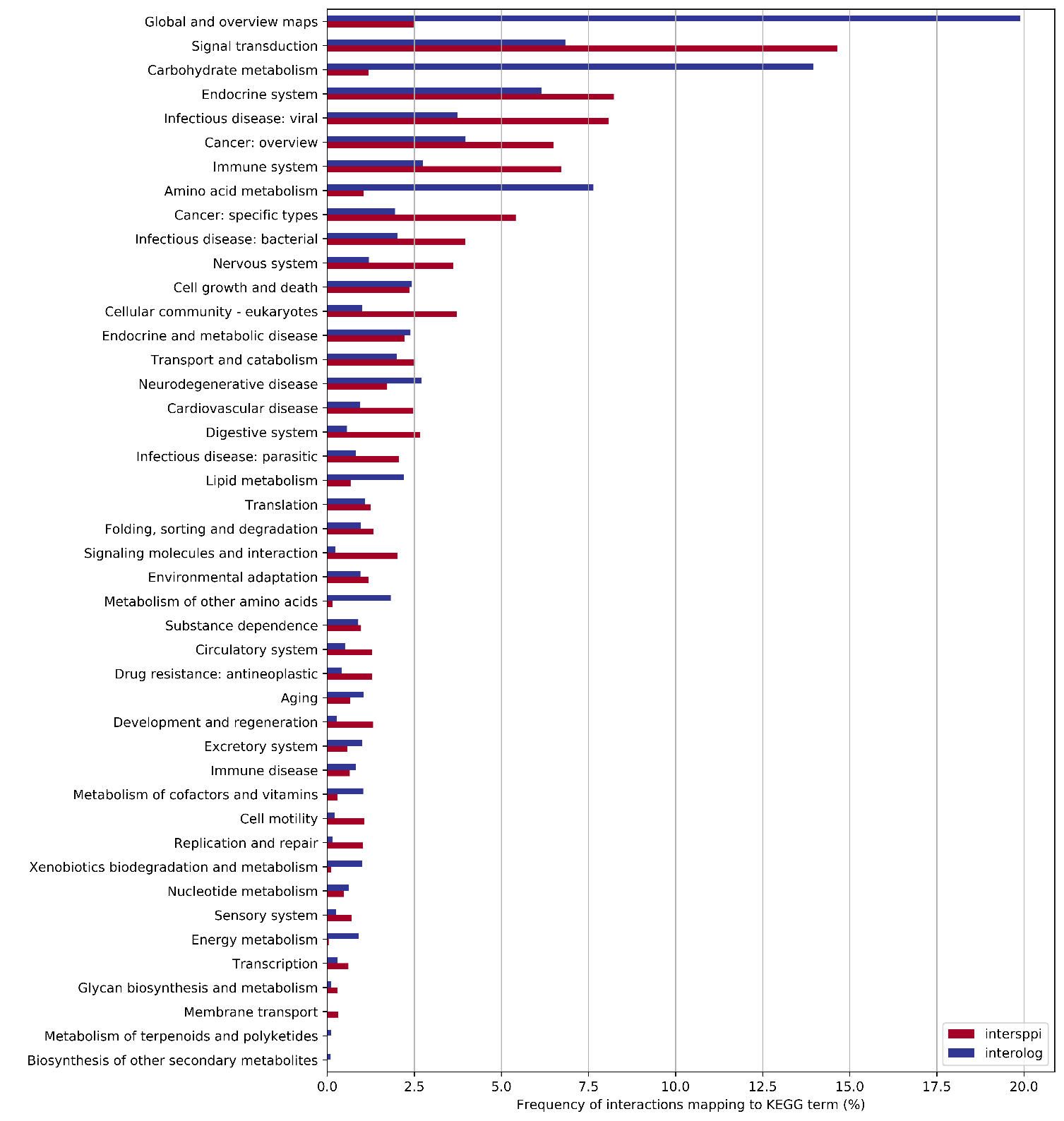
**

**Supplementary Figure 1.** Frequency of predicted interactions mapping to KEGG terms, according to prediction method.

**
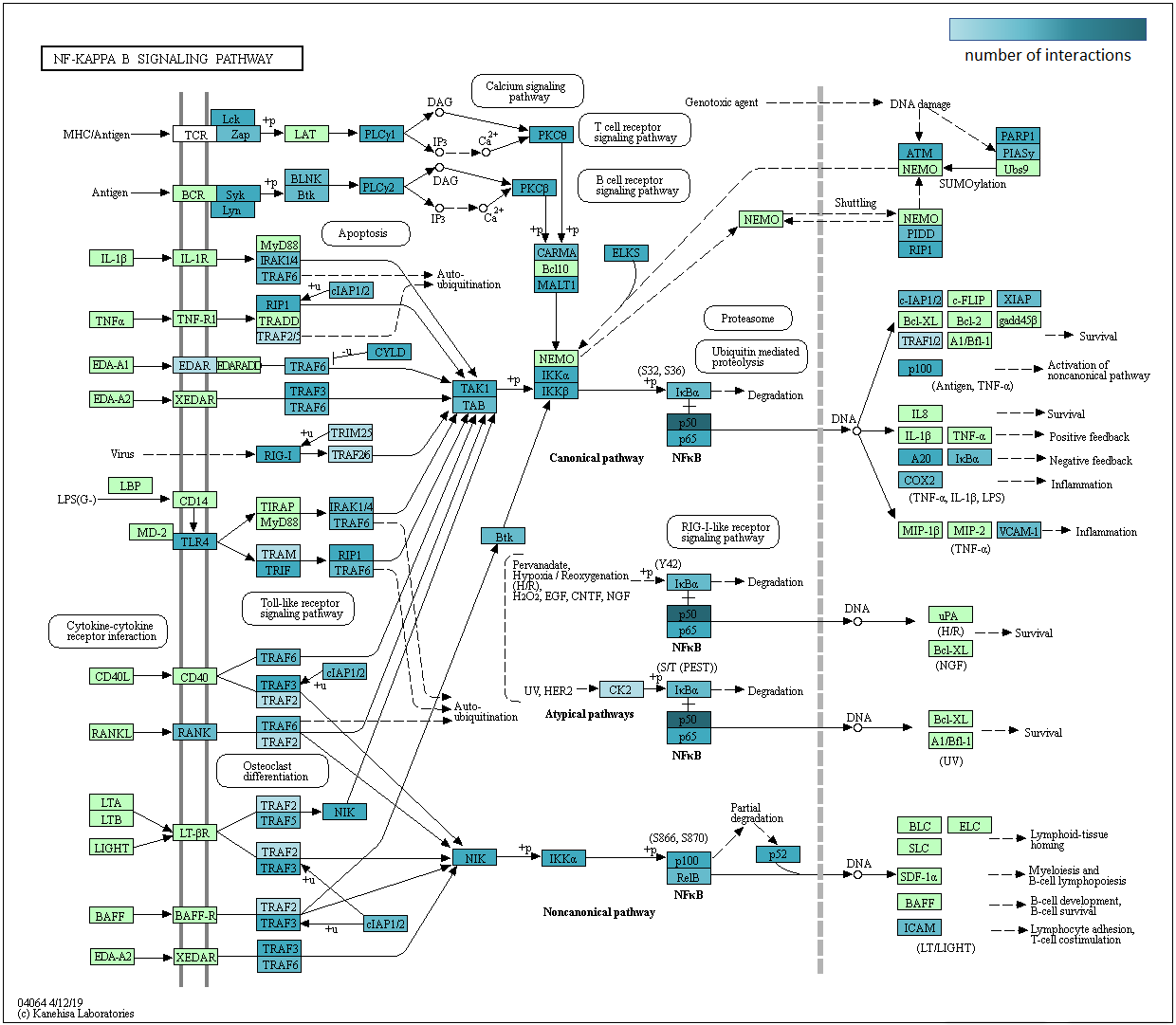
**

**Supplementary Figure 2.** Human KEGG NF-κB pathway, highlighting in blue the different human proteins predicted to interact with proteins from the EVs of *P. freudenreichii* CIRM-BIA 129. Non-interacting proteins are in green.

**
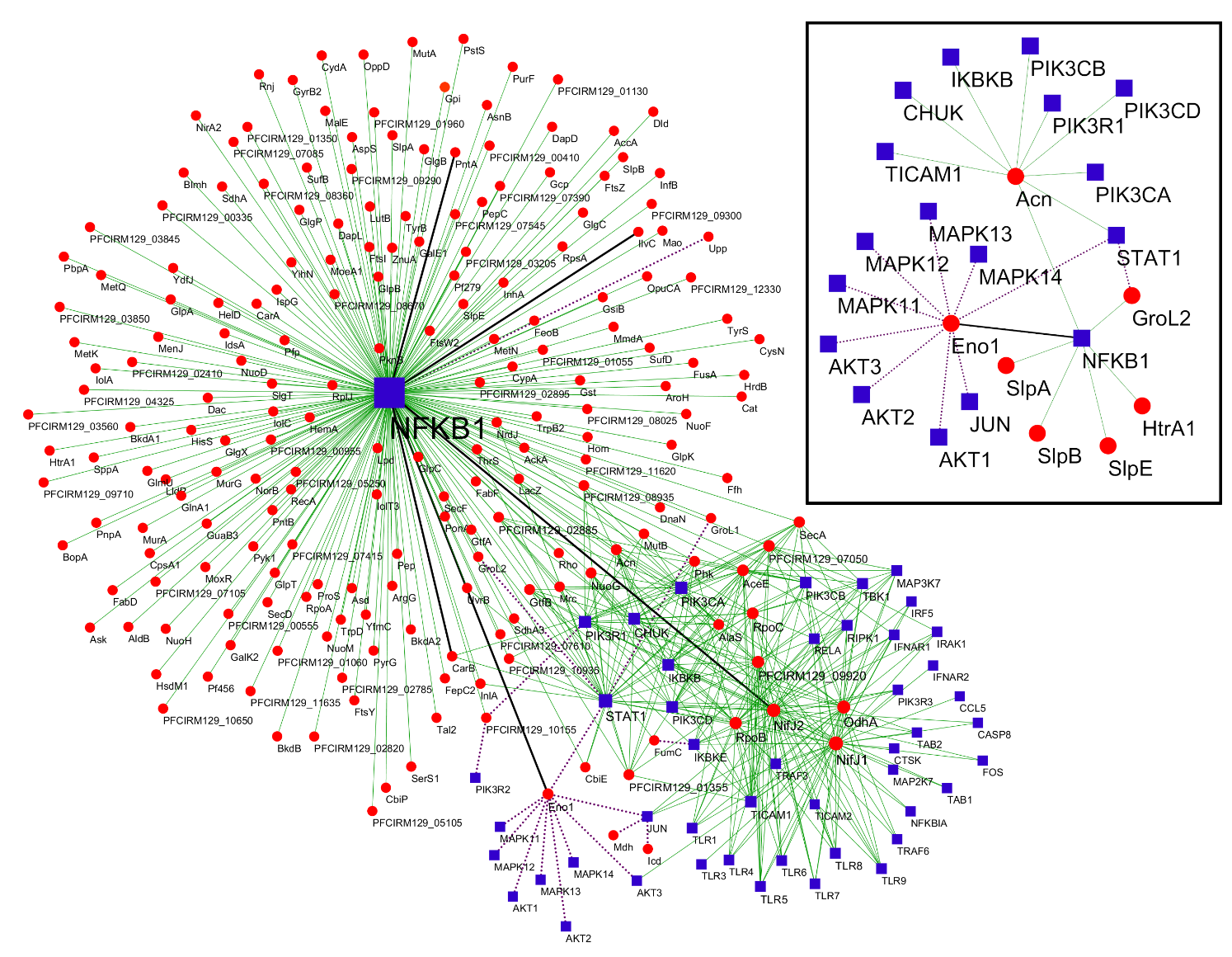
**

**Supplementary Figure 3.** Subnetwork of predicted interactions mapping to KEGG toll-like receptors (TLRs) pathway. Blue square nodes represent human proteins, red round nodes represent bacterial proteins, full thin green lines represent intersppi-predicted interactions, dotted purple lines represent interolog-predicted interactions and full thick black lines represent interactions shared by both methods. Inset: selected interactions involving bacterial proteins previously associated to immunomodulatory roles.
